# Letter to Editor: “The lncRNAs PART1 and ADAMTS9-AS2 act in an antithetic manner on AR signaling and induction of cellular senescence in prostate cancer cells”

**DOI:** 10.1097/JS9.0000000000002738

**Published:** 2025-06-12

**Authors:** Yongzheng Han, Yun Xu, Guangzhen Wu

**Affiliations:** a Department of Urology, the First Affiliated Hospital of Dalian Medical University, Dalian, China; b Department of Nursing, the First Affiliated Hospital of Dalian Medical University, Dalian, China

*Dear Editor,*

We are writing to commend the recent article by Mohammad Taheri *et al*, titled ***“The lncRNAs PART1 and ADAMTS9-AS2 act in an antithetic manner on AR signaling and induction of cellular senescence in prostate cancer cells***^[1]^***,”***published in the ***International Journal of Surgery***. Androgen receptor (AR) interact with multiple signaling pathways to promote the occurrence and progression of prostate cancer^[2]^. Therefore, clarifying the AR signaling mechanism is crucial for the treatment of prostate cancer. This study has made significant contributions to the field of prostate cancer research, enabling people to have a deeper understanding of the role of long non-coding RNAs (lncRNAs) in tumorigenesis. We hereby declare that this manuscript was translated and linguistic checked using artificial intelligence to ensure compliance with journal requirements. The TITAN Guideline checklist was submitted according to the requirements of the literature ***“Transparency In the reporting of Artificial Intelligence-The TITAN guideline”***^[3]^.

This study showed that PART1 acts as a co-repressor of AR signaling and ADAMTS9-AS2 as a co-activator. Specifically, in prostate cancer cells, supraphysiological androgen levels (SAL) up-regulate ADAMTS9-AS2 and down-regulate PART1. The authors showed that ADAMTS9-AS2 enhances AR activity and PART1 inhibits it. The opposite regulation of AR signaling by these two lncRNAs also affected cell senescence, with ADAMTS9-AS2 promoting Sal-induced senescence and PART1 inhibiting Sal-induced senescence in prostate cancer cells. This literature significantly enhances our understanding of the pathogenesis of prostate cancer and clarifies innovative treatment approaches. This work reflects the journal’s commitment to publishing transformative surgical science characterized by outstanding scientific precision and originality.

This study identified potential diagnostic markers and therapeutic targets by clarifying the effects of these lncRNAs on AR signaling, a key driver of prostate cancer development, which holds significant clinical significance. These findings may be especially beneficial for the treatment of advanced or castration-resistant prostate cancer. Additionally, the study provides a reliable platform for assessing tumor behavior and the effectiveness of specific treatments.

However, this groundbreaking research also raises further questions worthy of attention:

- How do the expression levels of PART1 and ADAMTS9-AS2 fluctuate as prostate cancer progresses from localized to metastatic stages? Clarifying this issue can reveal the optimal period for the intervention treatment of prostate cancer with lncRNAs.
- Could the author please elaborate on the potential advantages of combining lncRNA-targeted therapy with current androgen receptor pathway inhibitors (ARPIs)? Specifically, how might such combinations enhance the induction of cellular senescence and assist in overcoming resistance mechanisms in prostate cancer cells?
- Does targeting the ADAMTS9-AS2/hsa-miR-7-5p/CAV1 axis enhance SAL-induced drug-resistant tumor senescence? This study revealed this regulatory axis through bioinformatics analysis. Specifically, can the treatment targeting this axis exert a synergistic effect with bipolar androgen therapy and overcome the drug resistance mechanism by increasing the cellular senescence pathway?

Taheri *et al* clarified a key axis of AR signal transduction, paving the way for lncRNAs-targeted therapy of castration-resistant prostate cancer. We appreciate the author’s academic contributions and believe that these questions will support the further refinement of this promising research trajectory.

## Data Availability

The data that support the findings of this study are all included in this article.

## Research registration unique identifying number (UIN)

This study did not involve human subjects and was based on retrospective data analysis/literature review. Hence, research registration was not applicable.

## References

[1] Taheri M Schindler K Baniahmad A. The lncRNAs PART1 and ADAMTS9-AS2 act in an antithetic manner on AR signaling and induction of cellular senescence in prostate cancer cells. Int J Surg 2025;111:3646–64. 40143747 10.1097/JS9.0000000000002334 PMC12165499

[2] Quintero JC Díaz NF Rodríguez-Dorantes M Camacho-Arroyo I. Cancer stem cells and androgen receptor signaling: partners in disease progression. Int J Mol Sci 2023;24:15085. 37894767 10.3390/ijms242015085 PMC10606328

[3] Agha RA Mathew G Rashid R. Transparency in the reporting of artificial intelligence – the TITAN guideline. Prem J Sci 2025;10:100082.

